# Diseases of Women

**Published:** 1920-09

**Authors:** 


**Diseases of Women.** By Ten Teachers. Under the direction of Comyns Berkeley, M.A., M.D., M.C. Edited by Comyns Berkeley, H. Russell Andrews and J. S. Fairbairn. Pp. xi., 650. London: Edward Arnold. 1919. Price 30s. net.

This work will form a welcome addition to the student's library of gynæcological text-books. The collaboration of ten gynæcologists at present engaged in teaching is unique, but in this particular instance is eminently successful. The danger of indefinite statements, which is always likely when several writers collaborate, has been avoided, and the result is a very sound and practical guide to the student and the busy practitioner. The description of the anatomy and the development of the female pelvis is careful and accurate without being overloaded with detail. The plates are good, and the sections relating to the varieties of affections and methods of diagnosis are clear and precise.

Under the head of Carcinoma statistics of several thousand cases from the records of the Middlesex Hospital are given, which form a striking illustration of the value and advantages of such statistics, especially as to the relative frequency of carcinoma of the cervix and of the body of the uterus, and its occurrence under different conditions in the parous and nulliparous. The necessity for education in the recognition of the symptoms on which early recognition of this disease depends is fully shown and the relative importance of the various symptoms is clearly stated. The different methods of palliative treatment in cases where radical operation is not possible are fully described, and remarks on the limitations of treatment by radium and X-rays are practical and sound.

The chapters on Urinary and Intestinal Disorders, including Acute Abdominal Affections and Acute and Chronic Appendicitis, are specially noteworthy, while the chapter on Neurasthenia and Functional Disorders is particularly worthy of study, especially the remarks on the numerous cases in which operative treatment is undertaken for the removal of symptoms, in many cases without definite physical signs.

The descriptions of the various operative procedures and of modern technique are both full and precise.

We consider that this volume will form a very valuable addition to the library of the senior student as well as of the busy general practitioner.

